# Efficacy of the immediate adipose-derived stromal vascular fraction autograft on functional sensorimotor recovery after spinal cord contusion in rats

**DOI:** 10.1186/s13287-024-03645-z

**Published:** 2024-02-02

**Authors:** Céline Ertlen, Mostafa Seblani, Maxime Bonnet, Jean-Michel Brezun, Thelma Coyle, Florence Sabatier, Stéphane Fuentes, Patrick Decherchi, Nicolas Serratrice, Tanguy Marqueste

**Affiliations:** 1 Aix-Marseille Univ, CNRS, ISM UMR 7287, Institut des Sciences du Mouvement: Etienne-Jules MAREY, Equipe Plasticité Des Systèmes Nerveux Et Musculaire (PSNM), Parc Scientifique Et Technologique de Luminy, Aix Marseille Univ, CC910 - 163, Avenue de Luminy, 13288 Marseille Cedex 09, France; 2 Assistance Publique - Hôpitaux de Marseille (AP-HM), INSERM 1409 Centre d’Investigation Clinique en Biothérapies, Unité de Culture Et Thérapie Cellulaire, Hôpital de La Conception, 147, Boulevard Baille, 13385 Marseille Cedex 05, France; 3 Assistance Publique - Hôpitaux de Marseille (AP-HM), Service de Neurochirurgie, Hôpital de La Timone, 264, Rue Saint-Pierre, 13005 Marseille, France

**Keywords:** Fat, Reflex, Stem cell, Cell therapy, Inflammation, Sensorimotor loop, Paraplegia, Neuroprotection

## Abstract

**Background:**

Spinal cord injuries (SCI) lead to functional alteration with important consequences such as motor and sensory disorders. The repair strategies developed to date remain ineffective. The adipose tissue-derived stromal vascular fraction (SVF) is composed of a cocktail of cells with trophic, pro-angiogenic and immunomodulatory effects. Numerous therapeutic benefits were shown for tissue reconstitution, peripheral neuropathy and for the improvement of neurodegenerative diseases. Here, the therapeutic efficacy of SVF on sensorimotor recovery after an acute thoracic spinal cord contusion in adult rats was determined.

**Method:**

Male Sprague Dawley rats (*n* = 45) were divided into 3 groups: SHAM (without SCI and treatment), NaCl (animals with a spinal lesion and receiving a saline injection through the dura mater) and SVF (animals with a spinal lesion and receiving a fraction of fat removed from adipocytes through the dura mater). Some animals were sacrificed 14 days after the start of the experiment to determine the inflammatory reaction by measuring the interleukin-1β, interleukin-6 and Tumor Necrosis Factor-α in the lesion area. Other animals were followed once a week for 12 weeks to assess functional recovery (postural and locomotor activities, sensorimotor coordination). At the end of this period, spinal reflexivity (rate-dependent depression of the H-reflex) and physiological adjustments (ventilatory response to metabosensitive muscle activation following muscle fatigue) were measured with electrophysiological tools.

**Results:**

Compared to non-treated animals, results indicated that the SVF reduced the endogenous inflammation and increased the behavioral recovery in treated animals. Moreover, H-reflex depression and ventilatory adjustments to muscle fatigue were found to be comparable between SHAM and SVF groups.

**Conclusion:**

Our results highlight the effectiveness of SVF and its high therapeutic potential to improve sensorimotor functions and to restore the segmental sensorimotor loop and the communication between supra- and sub-lesional spinal cord regions after traumatic contusion.

**Graphical Abstract:**

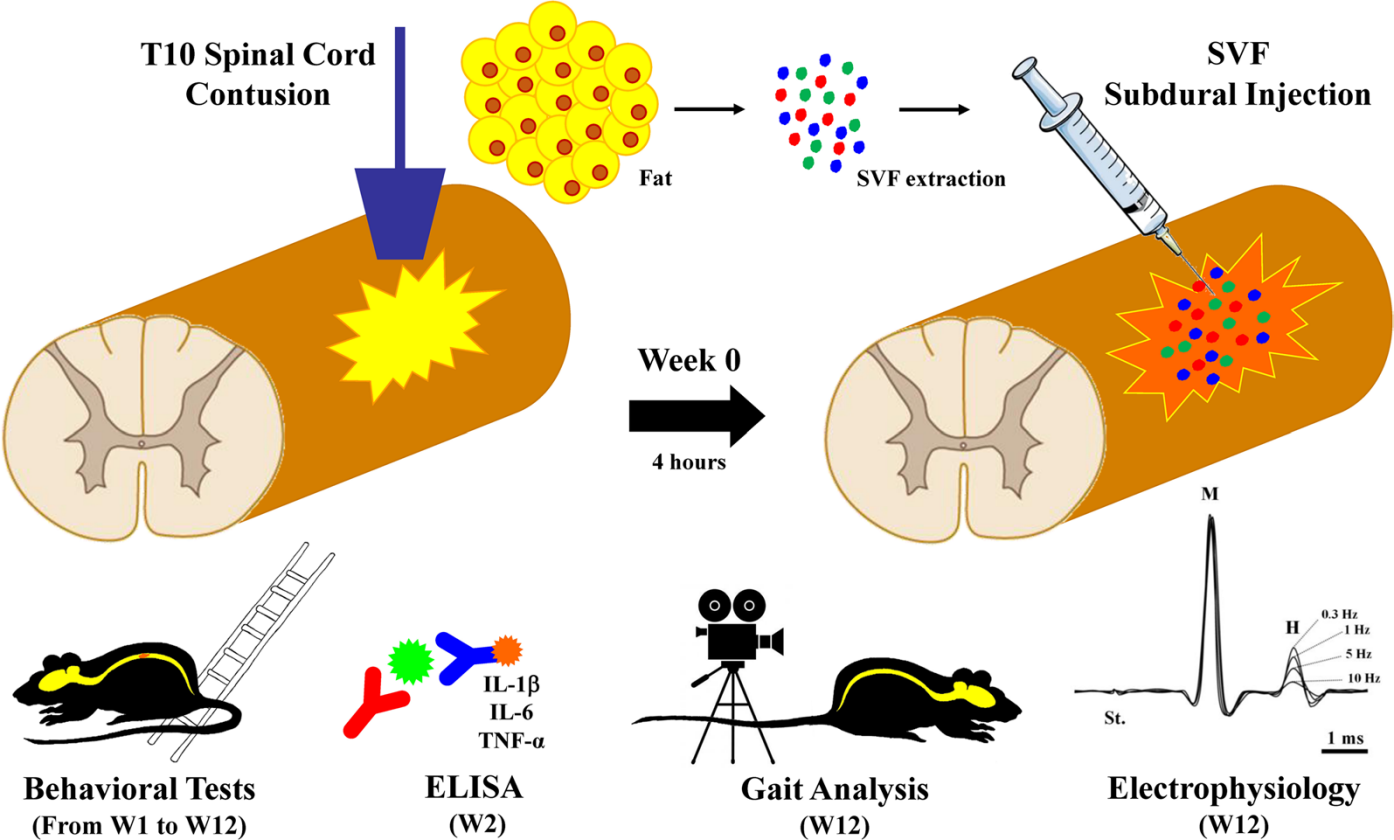

## Introduction

Traumatic spinal cord injury (SCI) is damage to the spinal cord tissue that can lead to lifelong disability. In human, lesion occurs after a violent impact on the spine due to motor vehicle accidents, acts of violence or recreational activities. Whatever the cause, injury leads to motor, sensory and autonomic dysfunctions below the level of traumatism with plegia, paralysis, multiple system dysfunction which can be subsequently associated with psychological alterations (excessive fatigue, feelings of tiredness, depressive states, poor-self efficacy, and anxiety) [1, 2]. Thus, SCI is a real health concern because of the decrease in life expectancy of the people concerned and the very high cost of care [3, 4]. Despite years of experimental research, numerous advances in the understanding of the pathophysiology of the spinal cord and the management of SCI, a plethora of promising experimental strategies and numerous clinical trials, today we are still unable to repair SCI and to propose a simple and effective repair strategy that can be applied to humans [5, 6].

Among non-pharmacological strategies, cellular therapies appear to be the subject of extensive study because of their potential ability to repair SCI through transplantation. Repair strategies have included various cell types such as neural stem and progenitor cells (NSPCs), oligodendrocyte progenitor cells (OPCs), mesenchymal stem cells (MSCs), Schwann cells, and olfactory ensheathing cells (OECs) [6–8]. Animal studies have shown that these cell types are good candidates because they induce functional improvement after SCI through several mechanisms such as neuroprotection, axon sprouting and/or regeneration, immunomodulation, and myelin regeneration [7, 8].

Adipose tissue is an endocrine organ capable of secreting a variety of hormones with numerous effects on overall metabolism. For example, the secreted adipokines have hypoglycemic and anti-inflammatory properties [9]. Through a minimally invasive procedure, the obtained lipoaspirated adipose tissue contains a high number of adipose-derived stem cells (ADSCs) or preadipocytes which can be easily isolated [10]. ADSCs are MSCs which seem to be of interest in tissue engineering and regenerative medicine. They have shown potential effects to treat autoimmune and neurodegenerative diseases, vascular and metabolic diseases, bone and cartilage regeneration, wound defects, and SCI [10–13]. Because ADSCs contain more somatic stem cells than bone marrow stem cells, their transplantation have demonstrated better effects in chronic and acute SCI than bone marrow stem cells transplantation [14, 15]. Indeed, several preclinical studies have highlighted their neuroprotective and regenerative potentials as well as their ability to improve sensorimotor recovery after SCI when injected or implanted with biomaterials directly into the lesioned cavity formed after injury or when infused intravenously [16–23]. Furthermore, in clinical studies, despite considerable heterogeneity among patients, overall, intrathecal injection was reported to be free of serious adverse effects, and motor and sensory ASIA scores and quality of life improved progressively over time [24, 25]. However, magnetic resonance imaging reveals no difference in the spinal cord damaged areas before and after the procedure [24]. Other human clinical trials are underway [13].

In addition to adult stem cells, fat is also composed of pericytes, fibroblasts, endothelial cells and its progenitors, smooth muscle cells, and a variety of immune cells such as adipose tissue macrophages and T regulatory cells [26]. Fraction of fat removed from adipocytes (adipose cells), connective tissue and blood constitutes the stromal vascular fraction (SVF). The characterization of the human SVF permitted to establish key markers for identifying the main cell populations with reliability. These populations consist of hematopoietic cells (monocytes/macrophages, neutrophils, and lymphocytes), ADSCs, endothelial cells, and preadipocytes [27]. Furthermore, it was reported that gender and anatomical site of sampling have no influence on the composition of the SVF [28]. This data suggests that the human SVF composition is uniform, regardless of these factors. Studies on the SVF composition in rats identified the same cell types [29, 30]. However, the proportion of these cells varies based on the SVF isolation method, whether derived from humans or animals [31]. Thus, it could be an alternative to the use of ADSCs. Indeed, a lot of studies suggest that the regenerative properties of lipoaspirate are found not only in the ADSCs but in the heterogeneous collection of cells contained in the SVF [32]. This mixture extracted from adipose tissue after digestion with collagenase has been successfully used to treat nerve lesion [32], multiple sclerosis [33, 34], diabetic foot ulcer and retinopathy [35–37], burn and radiation skin injuries [38–41], Crohn’s disease [42], ischemic heart failure [43, 44], bone regeneration [45, 46] and skin rejuvenation in aging process [47]. The SVF demonstrates significant angiogenic potential due to the existence of endothelial progenitor cells and their capability to secrete Vascular Endothelial Growth Factor (VEGF) [48]. After SCI, restoring the vascular network's integrity is vital for establishing a microenvironment conducive to regeneration at the lesion site. Studies on rats have demonstrated that administering VEGF to the injury site improves neovascularization following SCI. Moreover, this factor exhibits anti-inflammatory properties by triggering autophagic pathways, thereby improving neuronal viability following SCI [49, 50] Additionally, the SVF possesses the capability to mitigate neuroinflammation by activating both endogenous and exogenous leukocytes and macrophages. This leads to an elevation in the secretion of the anti-inflammatory interleukin-10 (IL-10), which in turn decreases the pro-inflammatory TNF-α [51]. The therapeutic potential of IL-10 has been demonstrated through several studies after SCI, as it can boost the numbers of macrophages and oligodendrocytes, promote neuronal survival, axonal regeneration, and myelination. Moreover, these studies revealed a locomotor function enhancement of rodents with SCI [52, 53]. Finally, the potential of SVF for limiting neurodegeneration in an experimental autoimmune encephalomyelitis (EAE) model has been highlighted [51] and the capacity of ADSC to differentiate in neurons. Thus, due to its angiogenic, immunomodulatory, differentiating, regenerating, and healing potential and the extracellular matrix components it contains [54], we hypothesized that SVF would provide benefits in an experimental model of acute spinal cord contusion in rats. The specific focus here is to evaluate functional sensorimotor recovery and to investigate the integrity of engaged neural networks.

In the present study, we injected, through the dura mater, within four hours post-injury, 50 µl containing 10^6^ cells of autologous adipose-derived SVF, into the 10th thoracic vertebra (T10) contused spinal cord to limit the functional deficits that develop after the initial injury and to elicit post-injury recoveries. Treated rats were compared to control (without SCI) and to lesioned animals that received a saline solution after SCI. BBB locomotor rating scale, gait analysis and ladder rung climbing tests performed once a week before lesion (PRE-) and for three months (from week 1 to 12) provided information on sensorimotor recoveries. The sacrifice of additional animals at two weeks post-injury allowed the evaluation of the inflammatory reaction (i.e., when the peak of the pro-inflammatory mediators has passed) [55]. Finally, electrophysiological recordings of H-reflex and ventilatory adjustments to muscle fatigue were used to assess the functioning of the sensorimotor loops under the lesion and their control by supraspinal levels.

## Materials and methods

### Animals

Experiments were performed on 45 adult male Sprague Dawley rats, weighing between 250 and 300 g (Élevage JANVIER®, Centre d’Élevage Roger JANVIER, Le Genest Saint Isle, France), hosted two per cage in smooth-bottomed plastic cages in a laboratory animal house maintained on a 12:12-h light/dark photoperiod and at 22 °C. Drinking water and rat chow (Safe®, Augy, France) were available ad libitum. Animals were housed in the animal facility during 2 weeks before the initiation of the experiment. This habituation period, during which animals regularly practiced the behavioral tasks, allowed to decrease inter-individual differences and to reach optimal performances. At the end of this period, PRE- values for each test (BBB-test, gait analysis and ladder climbing test) were recorded.

### Ethical considerations

Experiments were conducted according to the French legislation (Decrees and orders N°2013–118 of February 1st, 2013, JORF n°0032) concerning animal care guidelines on animal experimentation, and after approval by animal Care Committees of *Aix-Marseille Université* (AMU) and *Centre National de la Recherche Scientifique* (CNRS). The authorization number granted by the French Ministry of Higher Education, Research, and Innovation (MESRI) is APAFIS#30,957. All persons are licensed to conduct live animal experiments and all experimental rooms have a national authorization to accommodate animals (License n°B13.013.06). Furthermore, experiments were performed in accordance with the recommendations provided in the Guide for Care and Use of Laboratory Animals (U.S. Department of Health and Human Services, National Institutes of Health), with the directives 86/609/EEC and 010/63/EU of the European Parliament and of the Council of 24 November 1986 and of 22 September 2010, respectively, and with the ARRIVE (Animal Research: Reporting of In Vivo Experiments) guidelines.

The health status of the animals was daily controlled and any animals presenting signs of suffering such as screech, prostration, hyperactivity, significant weight loss (15 to 20%) and paw-eating behavior were sacrificed.

After surgery, animals were also kept under a heat lamp until thermoregulation was reestablished. They received subcutaneous injections (2 ml) of saline to replace fluid lost during the surgical procedure two to three times per day until they started to drink alone. Buprenorphine (0.03 mg.kg^−1^, Bruprécare® Multi-dose, Axience Santé Animale SAS, Pantin, France) was subcutaneously given daily for 3 days and a wide spectrum antibiotic (Oxytetracycline, 400 mg.l^−1^, Sigma Aldrich, Saint-Quentin Fallavier, France) was dissolved in the drinking water during 1 week to prevent any infection. If necessary, manual bladder expression was performed at least twice daily and nutritional supplement (Nutri-Plus Gel®, Virbac®, Carros, France) was given for weight loss. Postoperative nursing care also included visual inspection for skin irritation or decubitus ulcers and cleansing the hindquarters with soap and water followed by rapid drying of the fur with a bath towel.

### Protocol design and experimental groups

After two weeks familiarization (1 h per day, 3 days per week) on an open-field (BBB-test), the walking corridor (gait analysis) and the ladder (ladder climbing test), and after measurement of the reference values (PRE-) for each behavioral test, animals were randomly assigned into 3 experimental groups: (1) SHAM (*n* = 15) in which a surgery without spinal lesion was performed, (2) NaCl (*n* = 15) in which a thoracic T10 contusion was performed (W0) followed by an immediate injection of NaCl into the lesion area through the dura, (3) SVF (*n* = 15) in which epididymal fat was harvested, treated to extract SVF and autologously injected into the lesion area through the dura. In order to comply with the 3Rs (Replacement, Reduction and Refinement) ethical rule on the number of animals required to show a significant difference, the sample size per group was determined based on the first BBB results obtained at W12 from rats in the NaCl and SVF groups (3 rats in each group) where a common standard deviation (SD) of 2.92 were calculated and with a first-species risk α of 0.05 and a 1 − *β* power of 0.9 (epiR package 0.9–96 and two tailed test), the sample size is found to be 10 rats in each group.

**Fig. 1:**
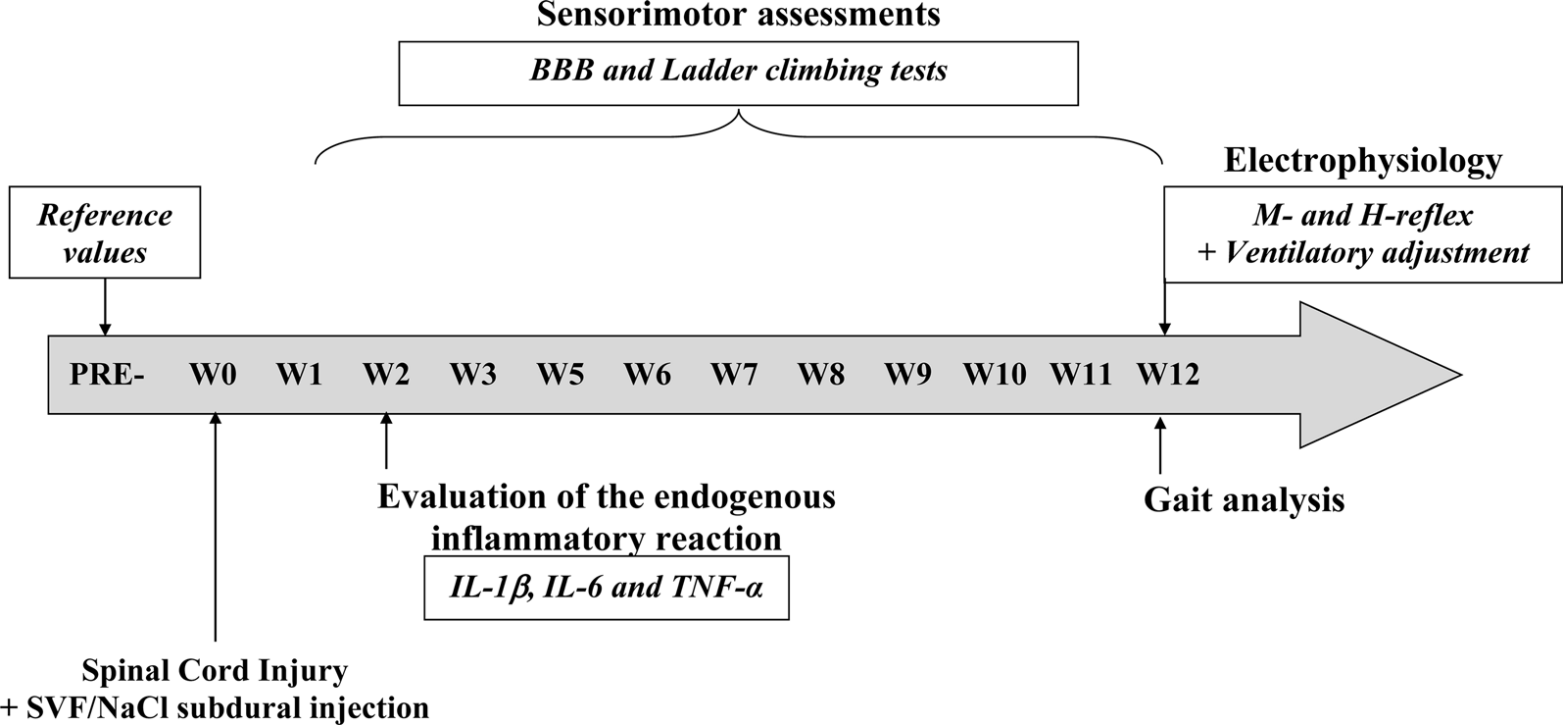
*Experimental protocol.* One week before the surgery, baseline values (PRE-) for each functional test were recorded. The surgery and injections of NaCl and SVF were performed at W0. Subsequently, sensorimotor recoveries were measured once a week for 12 weeks (from W1 to W12) using behavioral tests (BBB and Ladder climbing tests). The endogenous inflammatory response was analyzed at W2 (2 weeks after the injury). At the end of W2, gait analysis test was performed, and electrophysiological tests recorded the M-wave and the H-reflex

Fifteen animals of the SHAM (*n* = 5), NaCl (*n* = 5) and SVF (*n* = 5) groups were sacrificed two weeks (W2) post-injury to evaluate the endogenous inflammation at the lesion site. For other animals (*n* = 30), sensory and motor recovery in the hindlimbs was measured once a week, one week before the injury (PRE-) then from one week after the injury (W1) throughout the twelve subsequent weeks, by using the behavioral tests (BBB and ladder climbing tests). Then, twelve weeks (W12) post-injury, gait analysis test was performed, and sensorimotor loops were evaluated using electrophysiological recordings of the M-wave and H-reflex below the lesion and after muscle fatigue activating medullary ventilatory center through metabosensitive pathway. The chronological order of our protocol is schematically shown in Fig. 1.

### Fat removal, SVF purification and characterization of its components

All surgeries were performed during the light cycle. For initial induction of anesthesia, animals from the group SVF were placed in an anesthetic chamber and isoflurane (Isoflo® 100%, Zoetis SAS, Malakoff, France), was delivered (3‐5% in oxygen). Then, they were deeply anesthetized by an intraperitoneal injection of a mixture containing ketamine (100 mg.kg^−1^, 100 mg.ml^−1^, Ketamine 1000®, Virbac®, Carros, France) and xylazine (10 mg.kg^−1^, 20 mg.ml^−1^, Xilasyn®2, Virbac®) and a dose of buprenorphine (0.03 mg.kg^−1^) was subcutaneously administered. Surgical procedures were performed in sterile conditions with the aid of binocular dissecting microscope (PZMIII®, World Precision Instruments Ltd, Hertfordshire, UK). During surgery, body temperature was maintained at 37 °C using a homeothermic feedback-controlled heating pad (Homeothermic Blanket Systems, Harvard apparatus Sarl, Les Ulis, France) and physiological constants (cardiac and ventilatory rhythms, pupil dilatation) were monitored to control the depth of anesthesia. Animals were positioned in dorsal decubitus, belly was shaved, disinfected (Vétédine® solution 10%, Vetoquinol S.A., Magny-Vernois, France) and a 1 cm midline ventral incision was performed through the abdominal wall to gain access to the abdominal cavity and to harvest around 1.3 ± 0.3 cm^3^ of epididymal fat. Muscles and skin were closed after laparotomy using polyglactine-910 suture (Vicryl® 3–0, Ethicon, Issy Les Moulineaux, France). The SVF sample was purified using a manual method [56]. The concentration of viable nucleated cells and cell viability percentage was determined using a LUNA-FX7™ (^ⓒ^Aligned Genetics, Logos Biosystem, France) in duplicate. The formula to determine the final concentration of SVF necessary to obtain 1 million cells in 50 µl of cell solution is as follows: Total number of living cells x volume of purified SVF (ml) × 0.05 (ml)/1000 (result in µl). The recovery yield corresponds to the total number of the SVF cells relative to the volume of fat. Finally, the SVF was packaged at room temperature to be injected within 4 h after the SCI.

### Spinal cord injury and SVF subdural injection

Only animals from NaCl and SVF groups were concerned. The surgery procedure leading to the exposure of the spinal cord was detailed previously [57]. Briefly, after anesthesia and an injection of buprenorphine (0.03 mg.kg^−1^), animals were positioned in ventral decubitus, back shaved, disinfected (Vétédine® solution 10%, Vetoquinol S.A., Magny-Vernois, France) and a midline dorsal incision was performed over the T6-T13 spinous processes. The superficial muscles were retracted using retractors to expose the thoracic vertebrae and a T10 dorsal laminectomy was performed to expose the spinal cord without affecting its integrity. The dura was left intact. Stabilization clamps were placed at the posterior processes of the vertebra T9 and T11 to support the vertebral column during impact.

The spinal cord was contused at the T10 level using an IH-0400 weight-drop impactor (Infinite Horizon Impactor, Precisions Systems and Instrumentation, LLC., Lexington, KY, USA) equipped with a 10-g rod with a flat circular impact surface of diameter 2.5 mm which detects rod velocity (displacement over time) and any impact-induced movement using digital optical potentiometers to calculate impact velocity and compression rate. The impact rod was centered above T10, slowly lowered until it contacted the dura, which was determined by completion of an electrical circuit that activated an auditory tone. Then, the cord was contused by dropping the rod from a height of 50 mm. The force applied through the impactor was 200 kdyn corresponding to a severe contusion [58]. The rod was dropped such that the impact velocity was of around 1.3 m/s.

After SCI, animals were maintained under anaesthesia for 4 h, time required to extract the SVF from the fat. Additional doses of anaesthesia were administered if necessary. Then, animals from the NaCl and SVF groups received, into the lesion site, a subdural injection of 50 µl NaCl and SVF, respectively, corresponding to an injection of 1 million of cells. This concentration was selected based on the literature. Indeed, several studies conducted in rats have demonstrated maximum efficacy of the SVF at a concentration of 10^6^ cells [38, 59, 60].

After injury, muscles were sutured in anatomical layers and the skin was closed (Vicryl® 3–0, Ethicon, Issy Les Moulineaux, France).

### Endogenous inflammation

Two weeks (W2) after the SCI, endogenous inflammatory reaction was evaluated at the lesion site. The detailed procedure had been described previously [57]. Briefly, after an initial sedation (isoflurane 3–5% in oxygen), animals were euthanized with a lethal dose of anesthetic (Pentobarbital sodium, 390 mg/kg, i.p., Euthasol® Vet., Dechra Veterinary Products S.A.S., Montigny-le-Bretonneux, France). A segment of spinal cord extending 5 mm rostral and caudal to the injury site was harvested, immediately immersed in isopentane, and stored at − 80 °C until further analysis. When all samples were collected, they were homogenized separately in 1 ml of Phosphate Buffer Sodium (PBS) for 30 s with a handheld homogenizer (Ika Ultra Turrax® disperser, Fisher Scientific SAS, Illkirch, France) equipped with plastic pestle tips which homogenize the tissue through vibrating motions and the obtained mixtures were centrifuged (centrifuge Sigma 2–16 PK Centrifuge Fisher Scientific SAS, Illkirch, France) for 12 min (12,000×*g*, 4 °C). A fraction (50 µl) of the supernatant containing soluble proteins was used to evaluate inflammation. The concentrations of Interleukin-1β (IL-1β), Interleukin- 6 (IL-6) and tumor necrosis factor-α (TNF-α) were measured using enzyme-linked immunosorbent assay (ELISA) kits containing specific antibodies (RAB0272, RAB0311 and RAB0480, Sigma Aldrich®, Saint-Quentin Fallavier, France) according to the instructions provided by the manufacturer, and all samples were run in duplicate. The absorbance was read at a wavelength of 450 nm using a microplate reader (Multiskan® Microplate Photometer, Thermo Fisher Scientific, Life Technologies SAS, Courtaboeuf, France). Concentrations were determined from a standard curve and based on the amount of tissue weighed before homogenization. Thus, the interleukins levels were expressed as pg/g of spinal cord.

### Behavioral tests

The day before surgery, reference values (PRE-) of each test were recorded. Then, for 12 weeks (once a week post-injury, from W1 to W12), the BBB and ladder climbing tests were performed once, and gait analysis data were collected at W12, by two experimenters blinded to treatment group.

*BBB-test.* Locomotor functions were measured using the Basso-Beattie-Bresnahan test [61]. Briefly, animals were placed on a circular Plexiglas® enclosure arena (95 cm diameter, 40 cm wall height) with an anti-slip floor. Once the animal walked continuously in the open-field environment, it was video-recorded using a camcorder (GoPro Hero11®, GoPro, Inc., San Mateo, CA, USA). The duration of the session was 4 min. Analysis, carried out later, was performed using the BBB-scale based on 22 levels of locomotor behavior (0: complete paralysis to 21: normal locomotion). For each rat, the locomotor scores of the two hindlimbs were averaged together to yield one score per test session.

*Ladder climbing test.* Fine sensorimotor coordination was tested during climbing a 45° inclined ladder (100 mm × 1500 mm). This task is an easily acquired spontaneous response that does not require conditioning (reward or compulsion). As previously described, this test was used to evaluate the capacities to correctly place the paw on round metal rungs (diameter: 4 mm spaced at equal intervals of 20 mm with 150-mm-high side walls) of a ladder during climbing [57, 62, 63]. Animals were placed at the bottom of the ladder and climbing was video recorded with a camcorder (GoPro Hero11®) from a position below the ladder so that the two hindlimbs were filmed with a ventral view. At the top of the ladder, animals had access to a dark box. Contrary to control animals climbing readily, with all four paws locating and grasping the rungs without fault, lesioned animals showed varying degrees of difficulty in locating the ladder rungs with the affected legs. Animals with SCI climbed the ladder using their forelimbs with the body weight support provided by the inclined position of the ladder. The video recordings were analyzed at slow-speed playback and the placing of the hindpaws over the rungs were scored as following: 0: the hindlimb was hanging in front of or behind the rungs and didn’t support climbing, 1: the hindlimb was used to support climbing but the hindpaw wasn’t placed correctly on the rung, and 2: the hindpaw was correctly placed on the rung and the position was maintained while the trunk and the contralateral limb were moving up. The scores obtained on each side were averaged. Thus, a climbing score ranging from 0 (without success, i.e., 0 grip with the hindpaws) to 2 (animal climbed 20 rungs of the ladder without faults, i.e., 20 grips with the hindpaws, 10 per leg) was calculated and normalized to the maximal score. The mean ratio obtained at each session was expressed as percentage of the mean ratio obtained at week 0 (PRE-).

*Gait analysis.* Quantitative assessment of motor function and coordination was performed with a home-made gait analysis system that consists of a long glass walking plate on a corridor (110 cm long, 8 cm wide, opaque walls 15 cm tall), a fluorescent light beamed into the glass plate and a camcorder (GoPro Hero11®) under the glass plate. The recordings were made in a dark environment. The light, reflected downward at each contact point, allowed the recording of the footprints on the walking plate. Gait analysis data were acquired only if the animals reached at least frequent weight supported stepping (BBB score ≥ 9) [64]. Animal performed unforced and uninterrupted crossing on the glass walkway at least three times. For each run, as previously described [65], average values were calculated for analysis for the following parameters:

- *Average speed*: speed of forward locomotion across the runway (cm.s^−1^).
- *Stride length*: distance between two successive placements of the same paw (cm).
- *Stance phase*: time of contact of the paw with the glass plate (s).
- *Swing phase*: time during which the paw is not in contact with the glass plate (s).
- *Step cycle*: time between two successive placements of a single paw: stance added to swing duration (s).
- *Phase dispersion*: evaluation of the synchrony of the initial contact between pairs of limbs. It indicates the timing between the first contacts of paw pairs (e.g., Right Forelimb-Left Hindlimb), expressed as a percentage of the step cycle of the reference paw. When the limbs are in phase, meaning they synchronize perfectly, the phase dispersion is 0%. However, when the limb pairs alternate, there is typically a phase dispersion of 50%, indicating that they are not synchronized.
- *Step sequence patterns*: determined as normal when the animal sequentially placed its four paws in an alternate, cruciate (diagonal sequences) or rotate (lateral sequences) pattern based on the order of limb recruitment [66].
- *Regulatory index (RI)*: degree of coordination among the limbs, expressed as a percentage of the number of normal step sequence patterns (NSSP) relative to the total number of paw placements (PP): RI = NSSP × 4/PP × 100 (%).

The gait analysis parameters were collected from five consecutive full steps for each animal and were analyzed using a bespoke function developed in the MATLAB R2022b (The MathWorks, Inc., Natick, MA, USA) environment based on MouseWalker system [67] and any inappropriately labeled prints were manually corrected frame by frame.

### Electrophysiological recordings

Twelve weeks post-injury (W12), after an initial sedation (isoflurane 3–5% in oxygen), animals were deeply anesthetized by intraperitoneal injection of a mixture containing ketamine (100 mg.kg^−1^, 100 mg.ml^−1^, Ketamine 1000®, Virbac®) and xylazine (10 mg.kg^−1^, 20 mg.ml^−1^, Xilasyn®2, Virbac®), and prepared for electrophysiological recordings as previously described [68–70]. Briefly, the *peroneal* nerves from both hindlimbs were dissected free from surrounding tissues for stimulation. Then, the *Tibialis anterior* muscles from both hindlimbs were exposed for electromyographic (EMG) recording.

*M- and H-waves.* The M- and H-waves were recorded after electrical stimulation of the *peroneal* nerve. As previously described, in the first step, the rate-dependent depression (RDD) of the H-reflex also called rate-sensitive depression (RSD), frequency-related depression (FRD), frequency-dependent depression (FDD) and low-frequency depression (LFD) (i.e., the decrease in reflex magnitude relative to repetition rate) was analyzed by expressing the *H*_max_/*M*_max_ ratio obtained at the stimulation frequency of 1, 5 and 10 Hz to the *H*_max_/*M*_max_ ratio obtained at the baseline frequency of 0.3 Hz [57, 68, 69, 71–74]. In the second step, the *M* and *H*-reflex responses to repetitive stimulation at 10 Hz (0.5 ms, 5 × threshold) were elicited. Then, *H*-reflex amplitude of the second, third, fourth and fifth responses were measured from the average of five trials and *H*/*M* ratios (2nd, 3rd, 4th, 5th) were expressed as a percentage of the first *H*/*M* ratio (1st), also averaged over five trials [72].

*Physiological reflexes.* Ventilatory adjustments, recorded through the canula inserted into the trachea, were measured during and after activation of metabosensitive afferent fibers by *Tibialis anterior* muscle repetitive stimulation and under regional circulatory occlusion which isolated and maintained the neural drive as well as abolishing the hormonal communication [75]. Without SCI, a 3 min 10 Hz muscle stimulation induces a muscle fatigue that activates the metabosensitive afferents and subsequently an increase in the frequency and amplitude of ventilation [75]. After a SCI, such stimulation is not fully effective, i.e., the ventilation response is diminished or abolished, depending on the size of the lesion and the ascending pathways spared. As previously described, this test can be used to evaluate the therapeutic effectiveness of a treatment [57, 68, 69, 72, 73].

Thus, changes in ventilatory activity during and after electrically-induced muscle fatigue (EIF) was expressed in percent [Δcycle/min (%)] of the mean cycles recorded two minutes before muscle stimulation.

#### Euthanasia

According to ethical recommendations, at the end of the electrophysiological recordings, animals were killed with an overdose of pentobarbital sodium (390 mg.kg^−1^, i.p., Euthasol® Vet.) and the spinal cord was removed for further analysis.

#### Statistical analysis

Analysis of ELISA data was performed with a non-parametric Kruskal–Wallis test and the results were expressed as median ± SEM. After checking their normal distribution, the data of each experimental group were compared using a software program (SigmaStat 14.0®, San Jose, CA, USA). A two-way ANOVA (groups factor x time or stimulation frequency factor) for repeated measures was used to compare behavioral scores from all groups and over time and to compare *H*_max_/*M*_max_ ratios from all groups. Then, statistics were completed with a multiple-comparison *post-hoc* test (Student–Newman–Keuls method). Data were expressed as mean ± SEM and significant difference was considered when *p* < 0.05.

## Results

### SVF composition

Analysis of the SVF indicated that cell viability was 92.8 ± 3.03% and that recovery yield was 1.67 ± 0.71 × 10^6^ cells per ml of fat. Furthermore, in group SVF, 10^6^ cells in the 50 µl was injected through the dura.

### Animals

After SCI, all animals exhibited dramatic and bilateral hindlimb paralysis with no movement or only slight joint movements. On the 45 operated rats, 15 rats from SHAM (*n* = 5), NaCl (*n* = 5) and SVF (*n* = 5) groups were sacrificed 2 weeks after the SCI for evaluation of the IL-1, IL6 and TNF-α levels. All other animals underwent the weekly behavioral tests to electrophysiological recordings. Their weight did not drop throughout the experiment.

### Endogenous inflammation

**Fig. 2:**
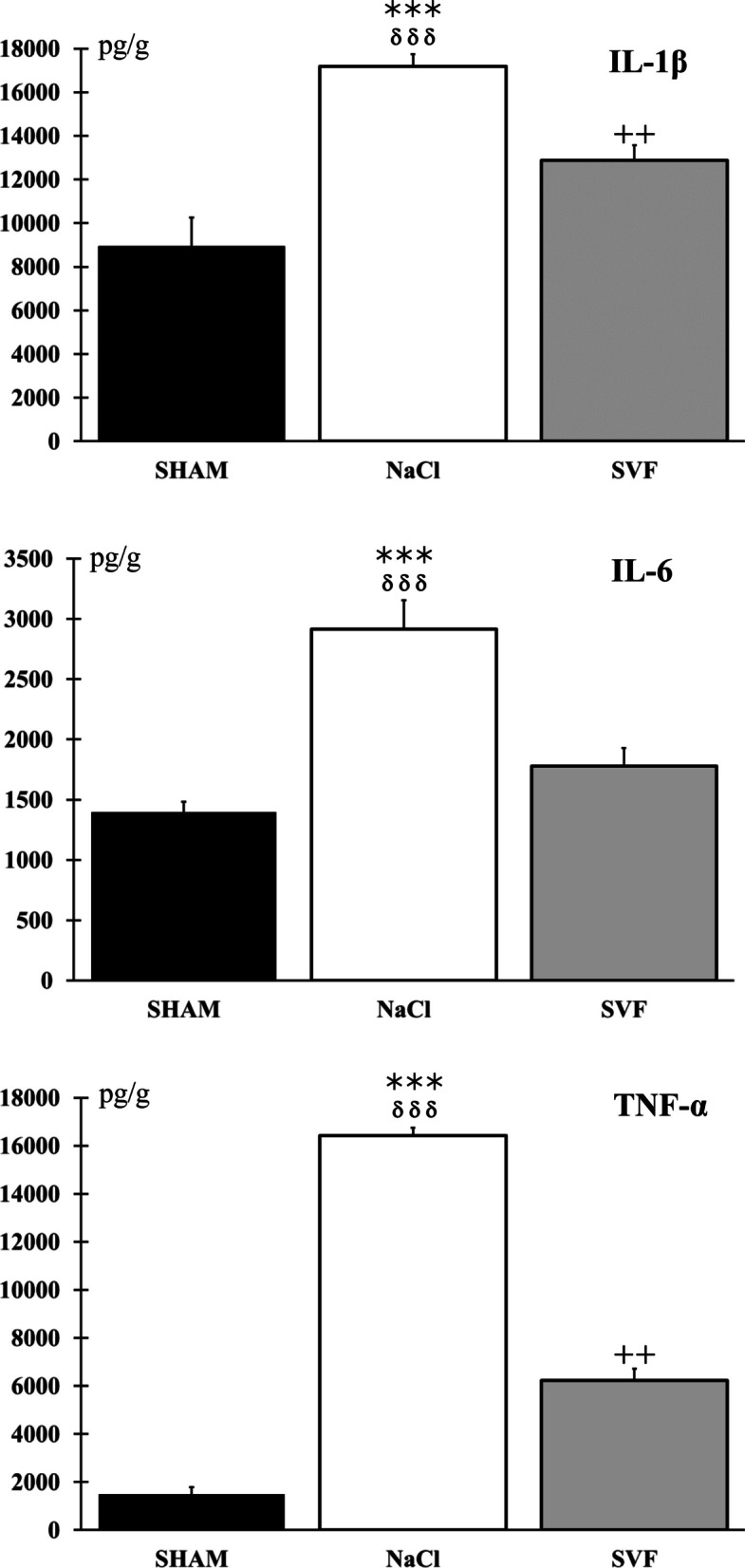
*Inflammatory reaction at the lesion site*. Comparison of IL-1β, IL-6 and TNF-α levels at the site of injury in the SHAM, NaCl and SVF groups, two weeks post-injury, indicated that the level of inflammation was higher in the NaCl group compared to the two other groups. Data also indicated that the inflammatory reaction was reduced in the SVF group, for the IL-6. Significant difference is indicated by a * (NaCl group *vs.* SHAM group), + (SVF group *vs.* SHAM group) and δ (SVF group *vs*. NaCl group). (2 symbols, *p* < 0.01 and 3 symbols, *p* < 0.001)

Measurement of IL-1β, IL-6 and TNF-α levels at the lesion site indicated that the level of inflammation was higher in the NaCl group than in the two other groups 2 weeks after the SCI. Data also showed that in the SVF group, the inflammatory reaction was reduced, notably, for the IL-6 in which no difference was observed between the SHAM and the SVF group (Fig. 2).

### Behavioral tests

**Fig. 3:**
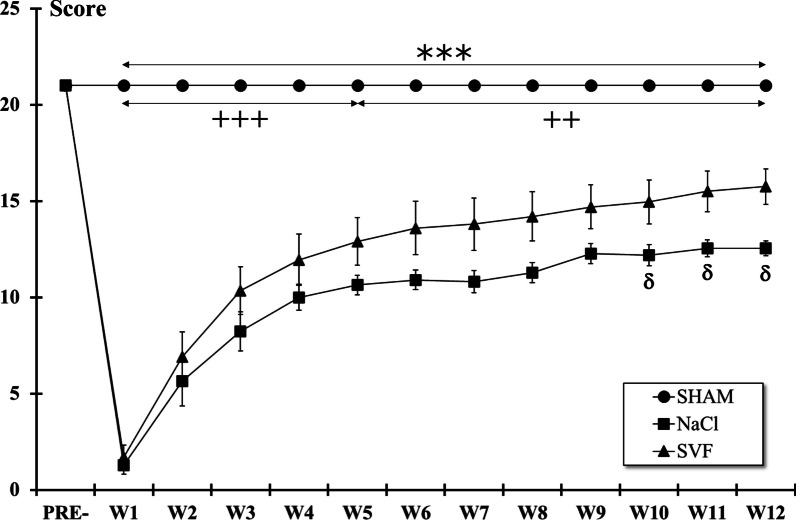
*BBB locomotor rating scale*. After the SCI, the BBB score in each injured group dropped significantly, and then a slow recovery was observed until W12. From W10, the BBB score in the SVF group became higher than in the NaCl groups. Significant difference in the BBB scores is indicated by a * (NaCl group *vs.* SHAM group), + (SVF group *vs.* SHAM group) and δ (SVF group *vs*. NaCl group). (1 symbol, *p* < 0.05, 2 symbols, *p* < 0.01 and 3 symbols, *p* < 0.001)

*BBB test.* Analysis of the BBB scores showed that scores significantly (*p* < 0.001) dropped one week (W1) post-injury in all lesioned groups (NaCl and SVF) compared to pre-injury values (PRE-). Although scores of these two groups remained statistically lower than the SHAM group over the course of the study, slow recovery was observed during the eleven following weeks reaching at W12 the score of 12.55 ± 0.38 (intermediate stage: intervals of uncoordinated stepping) for the NaCl group and 15.75 ± 0.92 (late stage: consistent forelimb and hindlimb coordination with consistent weight support) for the SVF group. Significant differences between NaCl and SVF groups were detected from W10 following injury (Fig. 3).

**Fig. 4:**
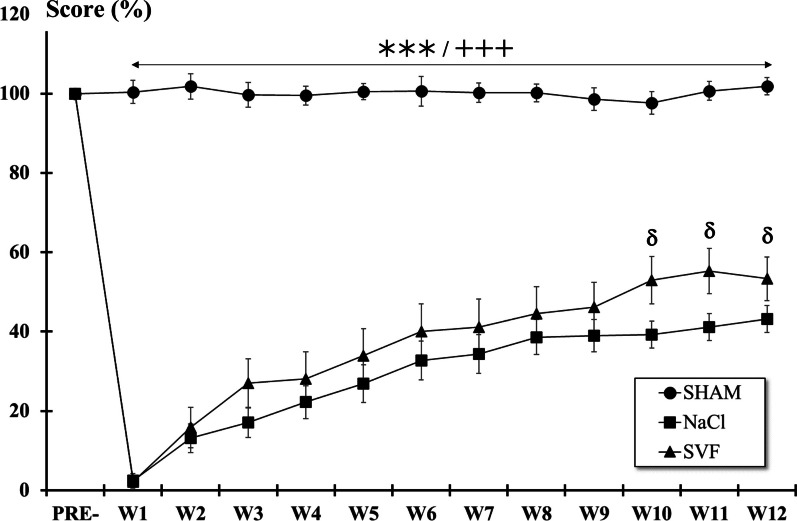
*Ladder climbing test*. After the SCI, the climbing score in each injured group dropped significantly, and then a slow recovery was observed until W12. From W10, some differences were observed between the NaCl and the SVF groups. Significant difference in the climbing scores is indicated by a * (NaCl group *vs.* SHAM group), + (SVF group *vs.* SHAM group) and δ (SVF group *vs*. NaCl group) (1 symbol, *p* < 0.05 and 3 symbols, *p* < 0.001)

*Ladder climbing test.* Immediately after the SCI, the climbing scores dropped significantly (*p* < 0.001). Then, a slow recovery was observed from W1 to W12 in the two lesioned groups (Fig. 4). However, although scores of these two groups remained statistically (*p* < 0.001) lower than the SHAM group over the course of the study, only significant differences (*p* < 0.05) between the two lesioned groups were observed for the three last weeks of the study.

**Fig. 5:**
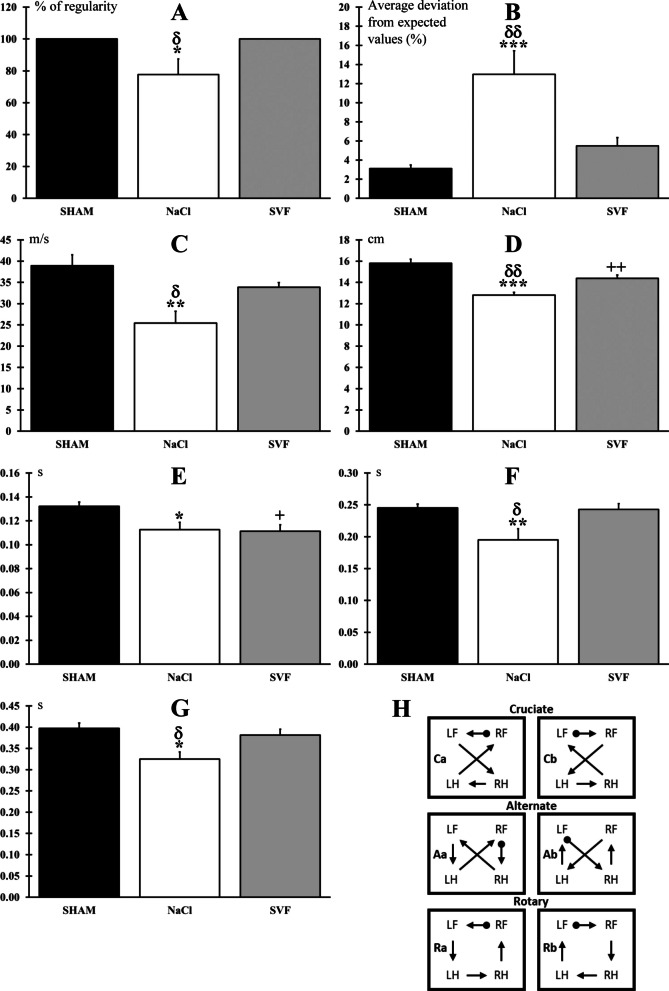
*Gait analysis parameters.* **A** Regulatory index. Animals treated with NaCl presented a significant decrease in the index of the degree of interlimb coordination compared to the SHAM and SVF groups. **B** Phase dispersion. Average deviation between hindlimbs from the expected value of 50%: animals treated with NaCl showed a significant increase compared to the SHAM and SVF groups. **C** Average speed. The average crossing speed on the glass plate was significantly reduced in the NaCl group compared to the two other groups. **D** Stride length. Average stride length between hindlimbs was significantly decreased in the NaCl and SVF groups compared to the SHAM group but to a lesser extent in the SVF group. **E** Swing phase. The average time of the swing phase between hindlimbs was significantly reduced in the NaCl and SVF groups compared to the SHAM group. **F** Stance phase. Average stance phase between hindlimbs showed a significant reduction in the NaCl compared to the two other groups. **G** Step cycle. Average step cycle between hindlimbs showed a significant reduction in the NaCl compared to the two other groups. **H** Step sequence patterns seen in normal rats (adapted from Cheng et al. [66]). Significant difference is indicated by a * (NaCl group *vs.* SHAM group), + (SVF group *vs.* SHAM group) and δ (SVF group *vs*. NaCl group) (1 symbol, *p* < 0.05, 2 symbols, *p* < 0.01 and 3 symbols, *p* < 0.001)

*Gait analysis.* At W12, the regulatory index was 100% in the SHAM and SVF groups, and significantly (*p* < 0.05) reduced in NaCl group (77.78 ± 0.9.68%) (Fig. 5A). Moreover, animals from NaCl (12.97 ± 6.96%) groups showed a significant (*p* < 0.001) increase in phase dispersion compared to the SHAM (3.12 ± 0.38%, *p* < 0.001) and SVF (5.49 ± 0.86%, *p* < 0.01) groups. No difference was found between the SHAM and SVF groups (Fig. 5B). Furthermore, there was no significant difference in the average crossing speed on the glass plate between the SVF (33.85 ± 1.08 cm.s^−1^) and the SHAM groups (38.95 ± 2.51 cm.s^−1^), while it was significantly reduced for the NaCl group (25.41 ± 2.82 cm.s^−1^) compared to the SHAM (*p* < 0.01) and SVF (*p* < 0.05) groups (Fig. 5C). Concerning the stride length, a significant reduction was observed in the NaCl (12.80 ± 0.26 cm) and SVF (14.38 ± 0.31 cm) groups (*p* < 0.001 and *p* < 0.01, respectively) compared to the SHAM group (15.79 ± 0.38 cm). However, this reduction was smaller in the SVF group, and a significant difference (*p* < 0.01) was found between the NaCl and the SVF group (Fig. 5D). For the swing phase time, a significant reduction (*p* < 0.05) was found in the NaCl (0.11 ± 0.01 s) and SVF (0.11 ± 0.01 s) groups compared to the SHAM group (0.13 ± 0.01 s): no difference was observed between the NaCl group and the SVF group (Fig. 5E). For the stance phase time, a significant decrease was found in the NaCl group (0.19 ± 0.02 s) compared to the two other groups (SHAM: 0.24 ± 0.01 s, *p* < 0.01 and SVF: 0.24 ± 0.01 s, *p* < 0.05) **(**Fig. 5F**)**. Finally, the step cycle time was significantly (*p* < 0.01) reduced in the NaCl group (0.32 ± 0.01 s) compared to the two other groups (SHAM: 0.39 ± 0.01 s and SVF: 0.38 ± 0.01 s) (Fig. 5G). The analysis of the frequency of regular step pattern showed a predominance of the “alternate” step pattern among all groups (97.78% for the SHAM group, 84.44% for the SVF group and 54.55% for the NaCl group). The “cruciate” and “rotate” step patterns, commonly used by normal animals, represented respectively 2.22 and 0% for the SHAM group, 2.22 and 13.33% for the SVF group, and 13.64 and 9.09% for the NaCl group **(**Fig. 5H**).**

### Electrophysiological recordings.

**Fig. 6:**
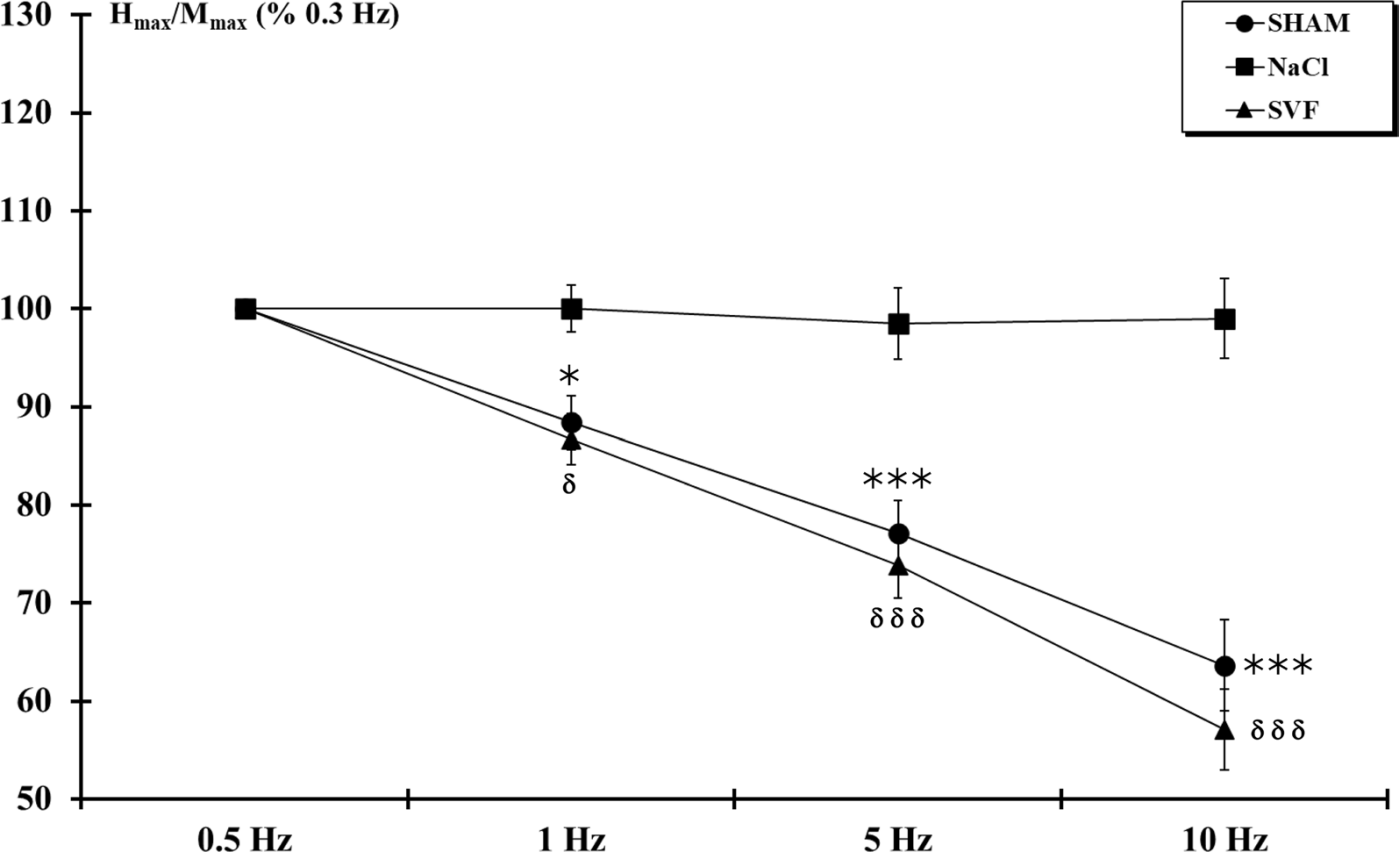
*H-reflex rate sensitivity*. *H*_max_/*M*_max_ ratio, measured after increasing the frequency of stimulation, showed, from 1 Hz stimulation, a significant depression in the SHAM and SVF groups. For a given frequency, significant difference in the *H*_max_/*M*_max_ ratio is indicated by a * (NaCl group *vs.* SHAM group) and δ (SVF group *vs*. NaCl group) (1 symbol, *p* < 0.05 and 3 symbols, *p* < 0.001)

The values of the *H*_max_/*M*_max_ ratios measured at the baseline stimulation (0.3 Hz) were 0.42 ± 0.03, 0.38 ± 0.03 and 0.40 ± 0.02 for SHAM, NaCl, and SVF groups, respectively. Although the ratio in the NaCl group seemed to be lower, data analysis indicated that these three ratios were not statistically different. However, except for animals from NaCl group, the H_max_/M_max_ ratio decreased when the frequency of stimulation increased (Fig. 6). Thus, in the SHAM group, the ratio values at 1, 5 and 10 Hz were 88.37 ± 2.71%, 77.08 ± 3.37% and 63.65 ± 4.60% of the ratio measured at the baseline stimulation, respectively, while in the SVF group, the values were 88.37 ± 2.60%, 73.86 ± 3.36% and 57.10 ± 4.10%, respectively. Data analysis indicated that, in the SHAM and SVF groups, the depression to stimulation statistically differed from the NaCl group at 1 Hz (*p* < 0.05), 5 Hz (*p* < 0.001) and 10 Hz (*p* < 0.001).

**Fig. 7:**
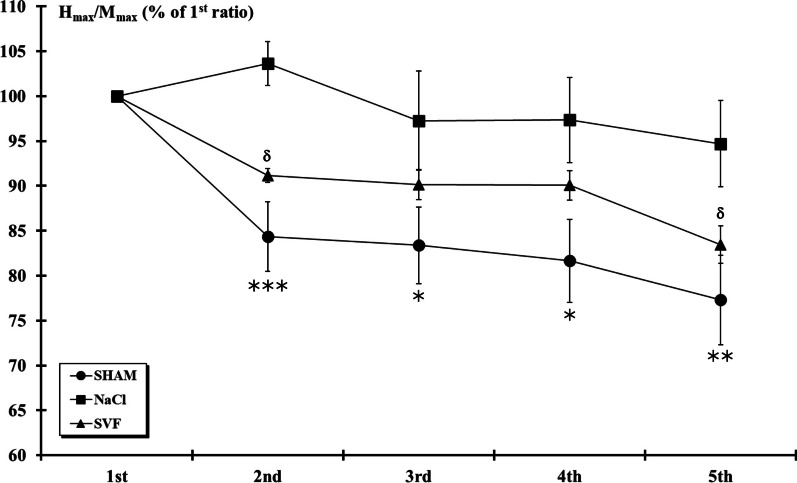
*H-reflex excitability*. At the 12th week post-injury, H-reflex responses at 10 Hz-train stimulation were markedly reduced by subsequent stimuli (normalized to 1st response) in the SHAM and SVF groups. This depression was absent in rats from NaCl group. For a given response, significant difference in the *H*_max_/*M*_max_ ratio is indicated by a * (NaCl group *vs.* SHAM group) and δ (SVF group *vs*. NaCl group) (1 symbol, *p* < 0.05, 2 symbols, *p* < 0.01 and 3 symbols, *p* < 0.001)

Analysis of M and H-reflex amplitude to repetitive stimulation at 10 Hz indicated that H-reflex responses were markedly reduced (*p* < 0.001) by subsequent stimuli (normalized to the first response) in the SHAM group (*p* < 0.001 for the 2nd, 3rd, 4th and 5th responses compared to the 1st response). Similar rate-sensitive depression was also observed in the SVF group but absent in NaCl group. Furthermore, significant (*p* < 0.05) differences were observed between the NaCl and the two other groups (Fig. 7).

**Fig. 8:**
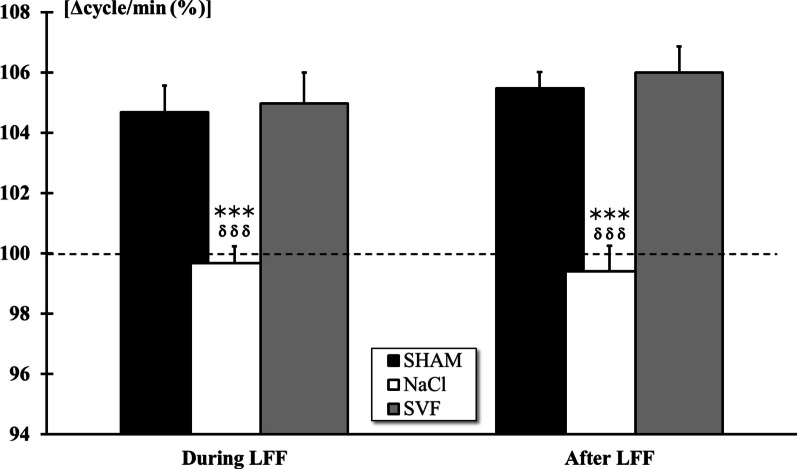
*Ventilatory adjustment to 3-min EIF*. The ventilatory activity significantly increased in the SHAM and SVF groups during and after 3-min muscle stimulation of the tibialis anterior muscle under local regional circulatory occlusion. Significant difference in the mean ventilatory frequency is indicated by a * (NaCl group *vs*. SHAM group) and δ (SVF group *vs*. NaCl group) (3 symbols, *p* < 0.001)

Analysis of ventilatory adjustments during and after activation of metabosensitive afferent fibers by *Tibialis anterior* muscle repetitive stimulation and under regional circulatory occlusion revealed (*p* < 0.001) a significant increase of ventilatory frequency only in SHAM and SVF groups. Furthermore, data analysis indicated a significant difference (*p* < 0.001) between NaCl and the two other groups. No difference was noted between SHAM and SVF groups (Fig. 8).

## Discussion

The aim of this study was to assess the effectiveness of early transplanting SVF in enhancing sensorimotor recovery following an acute thoracic spinal cord contusion in rats. Our hypothesis was that SVF, injected within the four hours after the T10 contusion, would modulate the immune response and improve sensorimotor recovery SCI.

Our results indicated that subdural injection of the SVF into the lesioned area reduced the inflammatory response two weeks after the injury and showed sensorimotor recovery over the 3-month analysis period. Furthermore, electrophysiological recordings taken twelve weeks after the injury showed the restoration of sensorimotor loop and modulation of the supraspinal network in the treated rats.

### The SVF reduced the SCI-induced inflammation level

Immediately after SCI, an inflammatory response occurs, characterized by a local secretion of pro-inflammatory cytokines [76] and an infiltration of immune cells [77–79]. This reaction may persist for several weeks and presents a peak of reactive astrocytes and macrophages around fourteen days after the injury [80]. This inflammatory response can lead to tissue degeneration and limit spontaneous regeneration.

The first step of our study was to demonstrate that the SVF did not increase endogenous inflammatory reaction more than without treatment, which could lead to further functional loss. To do this, we measured the levels of IL-1β, IL-6, and TNF-α at the site of the lesion. Our findings indicate that the SVF injection at the lesion site limits the inflammation. Indeed, in the SVF group, the pro-inflammatory cytokines level was significantly lower than in NaCl group, and no difference was found between SVF and SHAM group for IL-6.

Previous studies have demonstrated the anti-inflammatory and immunomodulatory properties of SVF, particularly through the attenuation of pro-inflammatory cytokine levels in the injured tissue and through the secretion of anti-inflammatory cytokines [81, 82]. Similarly, in spinal contusion in mice, the grafting of ADSCs at the injured site reduced the expression of inflammatory cytokines IL-1β, IL-6, and TNF-α. Furthermore, the authors also showed that the ADSCs administration inhibited the activation of the Jagged1/Notch signaling pathway, which is involved in neuroinflammation and normally increased after trauma [23]. ADSCs also promote M2 macrophages activation, which release IL-4, IL-10, and IL-13 anti-inflammatory cytokines, as well as immunomodulatory factors like TGF-β, that inhibit the action of pro-inflammatory immune cells and reduce the secretion of pro-inflammatory cytokines IL-1β, IL-6, and TNF-α. TGF-β additionally promotes the development of some subpopulations of regulatory T cells, which play an essential role in suppressing the immune response and preventing excessive inflammation [83, 84]. Additionally, VEGF secreted by endothelial progenitors present in the SVF can activate autophagy through the activation of autophagic proteins Beclin 1 and LC3B [85]. This helps to decrease neuroinflammation by eliminating cell flow. Thus, it can be hypothesized that the ADSCs and endothelial progenitors contained in the SVF exhibit similar action mechanisms that enable the reduction of the inflammatory levels at the site of the injury.

The specific mechanisms by which SVF reduces inflammation in the SCI microenvironment is still undetermined. Although the signaling pathways involved in the interaction between SVF and inflammatory cells remain unknown, it can be hypothesized that the modulation of the immune response and the reduction of pro-inflammatory cytokines could be attributed to the various cellular components contained in SVF, such as ADSCs and endothelial progenitor cells [86–88], and the mechanic support exerted by the extracellular matrix [54].

In our study, SVF demonstrated its ability to reduce inflammation in cases of acute SCI. However, further mechanistic investigations should be conducted to identify the involved metabolic pathways.

### The SVF improves sensorimotor recovery

The drop in sensorimotor scores in the contused rats was in accordance with previous studies demonstrating dramatic and significant deficits immediately after the SCI followed by a spontaneous recovery [57, 58, 89]. However, the BBB and the ladder climbing scores became significantly higher from the tenth week in rats treated with SVF compared to rats from NaCl group. Indeed, for the BBB test which evaluates the interlimb coordination, contrary to untreated rats that exhibited uncoordinated stepping, animals receiving SVF showed consistent forelimb and hindlimb coordination with consistent weight support. These results were confirmed with the ladder climbing test that evaluates the integration of fine sensory and motor inputs and provides an assessment of tactile sense, proprioception, and motor function.

Additionally, gait analysis revealed that rats treated with SVF had a faster locomotion, a higher stride length, stance phase and step cycle than NaCl group. One can notice that previous studies have described a correlation between gait speed and a shorter paw contact time with the ground [67, 90]. However, in our results, the shorter locomotion speed in the NaCl group can be explained by the increase in the number and decrease in the size of the steps. The absence of difference in the swing phase between the SVF and NaCl groups can be explained by the fact that during the gait cycle, the stance phase is longer than the swing phase and that its duration exhibits a lower level of variation in relation to speed [67]. The reduction in phase dispersion and the achievement of a maximum score on the regulation index indicate that SVF improves coordination during walking that is essential for ensuring smooth and efficient movement. It requires precise synchronization of limb movements and different body parts involved in walking. Altered coordination can result in an unstable gait, disordered movements, and increased energy expenditure. Thus, improvements observed in treated animals may allow higher stability, balance, and movement efficiency during walking. These results are consistent with the BBB scores obtained at 12 weeks after the SCI showing that treated animals displayed consistent coordination, highlighting a more precise motor control compared to non-treated animals that mostly exhibited uncoordinated steps.

Previous studies have shown that functional recovery after SCI following the use of ADSCs is correlated with reduced lesion volume, decreased glial scar, neural differentiation, and enhanced axonal outgrowth [15, 16, 91]. Given the shared components of the ADSC cocktail with that of the SVF, our observations may be associated with tissue preservation due to both neuroprotective and regenerative effects as shown by those studies. However, histological protocols are necessary to confirm this possible analysis. In a comparable experimental settings, the neuroprotection has been primarily attributed to the activation of the transforming growth factor-beta1 (TGF-β1)/ SMAD family member 3 protein (P-SMAD3)/procollagen-lysine,2-oxoglutarate 5-dioxygenase 2 (PLOD2) pathway in spinal cord neurons [15, 21]. This pathway regulates the synthesis and maturation of collagen, which is crucial for the structure and function of the extracellular matrix. In addition, the literature also reported that ADSCs implanted at the site of spinal injury in mice can differentiate into neurons and create functional neural circuits leading to the improvement in sensorimotor recovery [83]. ADSCs also secrete neurotrophic factors such as Nerve Growth Factor (NGF), Glial-Derived Neurotrophic Factor (GDNF), Hepatocyte Growth Factor (HGF), and Brain-Derived Neurotrophic Factor (BDNF) [92] that modulate the molecular pathways of PI3K/AKT and MAP kinase, and promote neuronal survival, growth, and differentiation [93]. In addition, endothelial progenitor cells contained in the SVF release VEGF which supports the survival of neurons, growth of neurites, and local network restructuring below the injury site leading to improvement of functional recovery after SCI in rats by activating the MAPK/Erk1/2 pathway [50]. Furthermore, the pericytes found in SVF possess significant regenerative abilities due to their abundance and function as a reservoir of stem cells that can be mobilized in case of injury. TGF-β controls their activation, differentiation, and recruitment to injured sites [94]. As a result, these various metabolic pathways can contribute to the regeneration of damaged nervous tissue and to the improvement of functional recovery, it is possible that the therapeutic effects observed in this study could be the result of the synergistic action of all these cells.

In our study, we observed the capacity of SVF to improve sensorimotor recovery after acute SCI. To understand the mechanisms underlying these improvements, histological analyses will be necessary.

### The SVF restores spinal and supraspinal sensorimotor loop

SCI affects neural networks both above and below the lesion. Indeed, depending on the severity of the injury, the sensorimotor loop can be directly interrupted, and/or ascending and descending pathways transmitting information from or toward these loops disrupted. This can lead to various sensorimotor impairments such as muscle paralysis, hypotonia, loss of reflexes below the level of injury, and spasticity [95]. It has been described that SCI results in an increase in H-reflex amplitude (and *H*_max_/*M*_max_ ratio) due to downward supraspinal inhibition release and a decrease in ventilatory adjustment to muscle fatigue due to upward excitatory interruption [57, 68, 69, 72, 73, 75, 96, 97].

Twelve weeks post-injury, our results showed a significant post-activation depression of the H-reflex in the SVF group, similarly to the non-lesioned group; in the non-treated group, the amplitude of the H-reflex remaining constant whatever the stimulation frequency. This result indicates that immediate administration of SVF after the injury was beneficial for the local neural network.Improvements also observed in the SVF group with 10 Hz stimulation trains could indicate a beneficial effect of the SVF on the supralesional ascending and descending pathways.

These findings are further supported by measuring the response of the ventilatory adjustments during the electrically-activation of metabosensitive fibers (groups III and IV afferents) originating from the *Tibialis anterior* muscle. Indeed, we observed that animals from the SVF group exhibited a similar ventilatory adjustment to that of the non-injured animals, whereas the NaCl group animals were unable to adjust their ventilatory frequency. This result could indicate that SVF has restored the transmission of neural impulses from the ascending pathway to the pontobulbar respiratory centers.

Understanding the mechanisms that underlie these functional recoveries is essential. Several studies have shown that ADSCs transplantation can lead to electrophysiological improvements after SCI. One study reported that the administration of ADSCs combined with a fibrin matrix after SCI in rats resulted in the restoration of post-activation depression of the H-reflex 60 days after the injury [98]. Additionally, a study demonstrated that ADSCs transplantation in rats with SCI resulted in significant recovery of motor-evoked potentials recorded at the spinal cord level [99]. This suggests that treatment with ADSCs contained in the SVF could restore the sensorimotor loop and spinal pathways after SCI suggesting neuroprotective or neuroregenerative properties of ADSCs.

Thus, it can be assumed that SVF, like ADSCs, restores the function of segmental neural networks and the transmission of information from these networks to supraspinal centers and vice versa. However, further additional research is needed to determine the potential of the SVF compared to ADSCs.

### The SVF is a good candidate for clinical trial

The accessibility, the minimally invasive liposuction procedure to extract SVF and its therapeutic potential make it a promising candidate for clinical trials in SCI treatment. Unlike ADSCs, the isolation of SVF does not require cell culture, eliminating the need for lengthy in vitro expansion. This direct isolation approach enables a faster and more convenient procedure, particularly in clinical settings. Moreover, the use of autologous SVF significantly reduces the risk of cell rejection, further enhancing its potential as a therapeutic option.

This study represents an initial exploration of the immediate administration of SVF and its effects on inflammatory response and functional recovery after SCI. Further investigations are needed to uncover the underlying mechanisms responsible of the observed improvements. In-depth histological analyses and investigations into the secretory molecules of different cell types within the SVF could provide additional insights into these mechanisms and the role of each of these elements.

Furthermore, our study does not provide insight into the fate of the cells from the SVF injected within the lesion. Pre-labelling the cells prior to injection could allow for the assessment of their viability and integration into the neural tissue. Furthermore, proper cell labelling would facilitate easier tracking of the cells and their interactions within the injured spinal cord.

### Conclusion

The accessibility, minimal invasiveness, autologous nature, and potential for functional improvement make SVF a promising candidate for clinical trials in SCI. However, although functional improvements were observed in animals treated with SVF, many questions remain as to the underlying mechanisms responsible for the reduced inflammatory response and functional recoveries. Understanding these mechanisms will help determine the full therapeutic potential of SVF and should enable us to propose a management and repair strategy adapted to patients with SCI.

## Data Availability

All data analyzed during this study are included in this publication. The datasets during and/or analyzed during the current study are available from the corresponding author on reasonable request.

## Abbreviations

ADSCs: Adipose-derived stem cells
EIF: Electrically-induced muscle fatigue
EMG: Electromyographic
FDD: Frequency-dependent depression
FRD: Frequency-related depression
IL-1β: Interleukin-1beta
IL-6: Interleukin-6
LFD: Low-frequency depression
MSCs: Mesenchymal stem cells
NSPCs: Neural stem and progenitor cells
OECs: Olfactory ensheathing cells
OPCs: Oligodendrocyte progenitor cells
P-SMAD3: SMAD family member 3 protein
PLOD2: Procollagen-lysine, 2-oxoglutarate 5-dioxygenase 2
RDD: Rate-dependent depression
RSD: Rate-sensitive depression
SCI: Spinal cord injury
SVF: Stromal vascular fraction
TGF-β1: Transforming growth factor-beta1
TNF-α: Tumor necrosis factor-alpha
T10: 10Th thoracic vertebra
